# Eco-friendly and sustainable approaches against aphids management (*Myzus persicae*) and dissipation studies of imidacloprid in important cash crop capsicum under protected and open condition

**DOI:** 10.1016/j.heliyon.2024.e34277

**Published:** 2024-07-25

**Authors:** Guru Peelapura Ningarajappa, Chidanand Shiveshankar Patil, Bhaidas Vitthal Deore, Yogesh Subhash Saindane, Rahul Kumar Anurag, Ashish Kumar Singh, Samir Barman, Tilak Mondal

**Affiliations:** a ICAR-Central Institute of Post-Harvest Engineering and Technology, Ludhiana, Punjab, 141004, India; b Department of Entomology, Mahatma Phule Krishi Vidyapeeth, Maharashtra, 413722, India; c ICAR-Vivekananda Parvatiya Krishi Anusandhan Sansthan, Almora, Uttarakhand, 263601, India; d ICAR-Indian Grassland and Fodder Research Institute, Jhansi, Uttar Pradesh, 284003, India; e ICAR-National Institute for Natural Fibre Engineering and Technology, Kolkata, 700040, West Bengal, India

**Keywords:** Capsicum, Aphids, Dissipation, Dietary exposure, Hazard quotient

## Abstract

Capsicum is generally infested with many biotic agents mainly sucking insects, among them the major is aphid (*Myzus persicae)*. Chemical management is one of the most common strategies for their management. However, there are no recommended insecticides for insect management in polyhouse. An experiment was designed to assess the bio-potency of four popularly used insecticides (Imidacloprid-17.8SL, Acephate-75SP, Dimethoate-30EC and Buprofezin-25SC), a botanical (Neem oil 10000 ppm) and two entomopathogenic fungi (*Metarhizium anisopliae* 1.15%WP and *Lecanicillium lecanii* 1.15%WP) for two consecutive seasons. Most effective and the highest reduction of aphid population (78.14–81.92 %) were found in imidacloprid (17.8SL) treated plots. This effective molecule imidacloprid was further studied for its dissipation pattern under polyhouse and open condition and found that the imidacloprid residues in capsicum fruit dissipated below quantification limit (BQL) within 10days after final spray and the residues in the soil sampled at harvest time were found below the detection level. The half-lives of imidacloprid were 1.88 and 2.61 days under polyhouse and 1.07 and 1.52 days in open field at recommended doses (25 g a.i. ha^−1^) and double doses (50 g a.i. ha^−1^) of application respectively. The dietary exposure of imidacloprid on capsicum fruit under both conditions exposed that hazard quotient (HQ) values obtained from the different treatment doses have not exceeded the upper limit of toxicity (HQ < 1) and imidacloprid residues in the fruits were found below the existing MRL (Maximum Residue Limit) values (0.5 mg/kg) at 3 days after its final applications. Thus, imidacloprid may be considered as the effective chemical management option against aphids in capsicum under polyhouse and open field having no harmful effect on human consumption.

## 1 Introduction

Capsicum (*Capsicum annuum* L. var. frutescens) popularly known as “Shimla Mirchi'', is among the most popular commercial polyhouse crops under the family of *Solanaceae*, which serve as powerhouse of many vital vitamins, minerals, amino acid and fibers and also have good sources of antioxidant properties [1]. India contributes almost one-fourth of the total capsicum production worldwide. It covered around 38 thousand hectares of land with the total production of around 5.63 lakh metric tonnes [2]. Capsicum is usually cultivated in both open fields as well as in polyhouse under controlled environmental conditions. Presently, in India its consumption is increasing gradually, especially by metropolitan consumers and there is an excellent demand for its export too because of its nutritional values [3]. In India the national productivity of capsicum is very poor as the crops suffer from multiple biotic stresses. More than 35 different insects and mite species are reported to cause damage in peppers, among them, sucking pests like thrips (*Scirtothrips dorsalis*), aphids (*Myzus persicae*) and mites (*Polyphagotarsonemus latus*) are important pests under polyhouse condition [4,5]. *Myzus persicae* are the most vital early invaders to the capsicum crop and difficult to manage as they are reported to be highly active throughout the year and it causes an estimated yield loss of around 50 t ha^−1^, when no chemical measure was taken [6]. Thus, to avoid the economic loss to the crop, chemical control became an inevitable management strategy against aphids.

Presently, in India insecticide formulations are registered and suggested by Central Insecticide Board and Registration Committee (CIB&RC) for use on green chilli against various targeting pests, but there are no such registered insecticides on capsicum crop under protected cultivation [7]. Therefore, farmers are mainly relying upon non-recommended conventional insecticides to get rid of aphids, which have led to the problem of pesticide residues. Imidacloprid [1-(6-chloro-3-pyridylmethyl)-N-nitroimidazolidin-2-ylideneamine], is one of the popular molecules in neonicotinoid group of new generation insecticide, widely used for controlling sucking insects such as aphids, termites, rice hoppers, white flies and ticks in all over the world. Imidacloprid shows as a nicotinic acetylcholine receptor (nAChRs) activity to over stimulation of the nerve cells which is responsible for paralysis and ultimately causes death of insects. It is the most popular broad-spectrum systemic insecticide because of having minute mammalian toxicity [8] and the safest agrochemicals registered in India for managing aphids in different vegetable crops [7]. Since capsicum is consumed afresh; residues are of concern which warns judicious use of pesticides. Recent reports revealed the occurrence of residues of non-recommended pesticides in the market and farm gate samples of capsicum [9]. Residual studies have become important to know the status of the food/fruit quality as it mainly interferes with the consumption, which directly affects the human diet [[10], [11], [12], [13], [14], [15]]. Keeping this in view, the current assignment was performed to evaluate the effectiveness of suitable insecticides along with biocontrol agents against aphids and economical production of capsicum with facts on the dissipation pattern of most effective insecticides in/on capsicum under polyhouse and open condition.

## 2 Materials and methods

### 2.1 Experiment location

Bio-efficacy trial was managed in the polyhouse at farmer's field at Kangoni, Maharashtra, India (19°23′50.1″N 74°52′23.5″E), for two *kharif* seasons (June to December). The seedlings of ‘*Bachata*’ (RijkZwaan India Ltd.) were transplanted on a broad bed and furrow (90 cm wide x 15 cm height x 45 cm inter row space) with a drainage space of 3m. All the cultivation operations were done according to standard management strategies given for that region. The trial was arranged within a Randomized Block Design (RBD) made up of three replications, each of the single replicates consisted of 70 plants. Natural infestation of aphids was recorded at regular intervals and the experiment on bio-efficacy was initiated after the population reached the economic threshold level (ETL) of 2 nymphs or adults per leaf. Insecticidal spray was not done prior to the treatment imposition.

### 2.2 Treatment details of bioassay

Initially a survey was conducted in Ahmednagar (Maharashtra), to decide the insecticides to be tested as treatments; in that randomly selected polyhouse capsicum farmers were interviewed using a structured schedule, to know their insecticide usage pattern for pest management in polyhouse capsicum. In reference to that, the commonly used insecticides, botanicals and biopesticides were finalized against aphids mentioned in Table 1. Insecticide application was done twice at 20 days interval.

**Table 1:** Details of treatments used for the management of aphid, *M. persicae* in capsicum.

Treatments	Treatment details	Dose
T_1_	Imidacloprid 17.8SL (Confidor®)	25 g a.i. ha^−1^
T_2_	Acephate 75SP (Starthene®)	584 g a.i. ha^−1^
T_3_	Dimethoate 30 EC (Rogor®)	300 g a.i. ha^−1^
T_4_	Buprofezin 25SC (Applaud®)	150 g a.i. ha^−1^
T_5_	*Metarhizium anisopliae*1.15%WP (1 × 10^8^ cfu/ml) (*Phule Metarhizium*)	2 g/L
T_6_	*Lecanicillium lecanii*1.15%WP (1 × 10^8^ cfu/ml) (*Phule Bugicide*)	2 g/L
T_7_	Neem oil-10000 ppm (Nimbicidine®)	5 mL/L
T_8_	Untreated control	–

Note: Sr. No. T1 to T4 and T7 were procured from local market. Sr. No. T5 and T6 the formulations were developed by Mahatma Phule Krishi Vidyapeeth, Rahuri (India). 400–800 L of water were recommended per hectare based on crop stage.

### 2.3 Observations

The number of adult or nymphs per leaf from six leaves of randomly tagged five plants was recorded. The observations were recorded at pre-treatment count (just prior application) and post-treatment count (7, 10 and 15 days after 1st and 2nd application). The mean survived aphid populations were worked out and used to calculate the mortality. Two computed values *i.e.*, per cent reduction over control (ROC) and per cent reduction over pre-treatment count (PTC) was done to know the performance of different insecticides. Fruits were harvested from each treatment separately and yield was recorded after each picking.

### 2.4 Statistical analysis

The statistical tool that was used here to conduct the experiment is the randomized block design. The significant effects of different chemical treatments over different stages (over different time intervals) have been studied through analysis of variance. The analysis was done by employing ‘*agricolae*’ package of R software.

### 2.5 Residue analysis/dissipation study

Based on the bio-efficacy results, the residue analysis and dissipation pattern of imidacloprid 17.8SL was further assessed. Field experiment for residue study of imidacloprid was also conducted at the above-mentioned place in capsicum following good agronomic practices under polyhouse and open field. The size of each treatment plot was 5m × 10m. The application of imidacloprid 17.8SL (Confidor) was given with the help of knapsack sprayer (15 L capacity) during fruiting period of capsicum after that next spraying at 10 days intervals. The experiment was executed in six treatments with four replications and randomized block design was followed for the statistical analysis. The treatments were consisted of recommended dosage of insecticide at the rate of 25 g a.i. ha^−1^ under polyhouse and open field (T1 & T4), double the recommended dosage at the rate of 50 g a.i. ha^−1^ (T2 & T5) along with untreated control plots at both conditions (T3 & T6) respectively.

### 2.6 Sampling for residual analysis

4 to 5 marketed size capsicum fruits were collected at different days (0 (2 h), 1, 3, 5, 7, 10 and 15 days) succeeding the final imidacloprid spray. The capsicum samples were harvested randomly from the different treatments, packed properly and shifted towards laboratory for further process and stored at −18 °C for a minimum period to avoid degradation. Soil samples were collected during the final harvest of the whole crop from the different sites of each treated plot at about 10–15 cm soil depth, dried properly, sieved and stored for future analysis.

### 2.7 Extraction and cleanup of imidacloprid from capsicum and soil sample

The QuEChERS analytical method [16,17] was adopted with slight modification for imidacloprid residue extraction and clean-up process from capsicum and soil samples. 10 g finely chopped fruit samples were placed in PP tubes (50 mL) along with acetonitrile (15 mL, HPLC grade) and homogenized the sample for 2–3min at 14,000–15,000 rpm. Next, 5 g of NaCl and anhydrous Na_2_SO_4_ were mixed on it and further centrifuged at 2500–3000 rpm for 2min. Cleanup process of extracted sample was done by using dispersive solid phase extraction (DSPE) method [18]. Afterwards, the acetonitrile solution was filtered using a 0.45 μm syringe filter before injection into the HPLC system. Similar method was adopted for residue extraction from the soil samples, where (2:1 v/v) acetonitrile: water solution was used for extraction.

### 2.8 Operating conditions of HPLC

Quantification and residual analysis of imidacloprid from capsicum and soil sample was executed using reverse phase high performance liquid chromatography (Shimadzu/HPLC-LC-20AT) with PDA detector and LC-solution software used for data analysis. Mobile phase was selected after several initial trails with mixtures of acetonitrile and Millipore water (Acetonitrile: Water = 80:20). The column (Eclipse XDB-C18; 4.6 mm × 150 mm; 5 μm particle size diameters) was equilibrated with the mobile phase at the flow rate of 0.8 mL/min, at 210 nm wavelength with the injection volume of 20 μL and the run time of 10min was used for the quantification of imidacloprid.

### 2.9 Preparation of standard solutions

The stock solution (1000 μg/mL) of imidacloprid (Sigma-Aldrich, Analytical grade) was prepared by dissolving 10 mg (±0.01 mg, purity 98 %) of standard in acetonitrile (HPLC grade) solvent in a volumetric flask (certified ‘A’ class) and kept at 4 °C in a refrigerator for future use. Further working solution (100 μg/mL) was made by diluting the stock solution, from which the standard curve was composed within the series from 0.05 to 1.0 μg/mL range by serial dilution.

### 2.10 Method validation

The current method was validated as per the standard guidelines of SANTE [17]. Different validations parameters of the imidacloprid standard were achieved in capsicum and soil matrices. The present study conducted several validation parameters, including linearity, trueness (recovery), precision, sensitivity, specificity, and matrix effect. Imidacloprid matrix-matched standards, ranged between 0.05 and 0.50 μg/mL concentrations were injected into the HPLC for finding the peak area. Linearity was judged based on calibration curve being made; specificity was calculated as percentages of average peak area of blank sample to the peak area of fortified imidacloprid standard in the blank matrix sample injected in five replications each. Recovery efficiency (RE) was measured by dividing detected average residues with the respective spiked level multiplied by 100. The repeatability of the developed method was estimated using relative standard deviation (RSD) values of each replicate of different fortified level. Intra-laboratory precision (reproducibility) was expressed as the Horwitz Ratio (HorRat) which calculated by following formula [19,20].

HorRat=RSD/PRSD

Where, PRSD represents predicted RSD = C^−0.15^ and C represents the concentration expressed as a mass fraction (10 ngg^−1^ = 10 × 10^−9^).

Sensitivity was measured in terms of limit of detection (LOD) and limit of quantifications (LOQ) of the system which were estimated by considering the corresponding value of signal to noise ratio of 3 and 10. Matrix effect (ME) and process efficiency (PE) were calculated employing the established formula [21].

ME(%)=[peakareaofpost‐extractionspiking/peakareaofthesolventstandard]x100

PE(%)=[MExRE]/100

#### 2.10.1 Dissipation of imidacloprid

Dissipation pattern of imidacloprid was studied in capsicum crop under open and polyhouse at two treatment levels i.e., recommended dose (@ 25 g a.i.ha^−1^) and double the recommended dose (@ 50 g a.i.ha^−1^). Dissipation of imidacloprid is performed to first order kinetics [8] and the dissipation kinetics of imidacloprid were obtained using the equation

$$Ct=\log\;C_{0}+Kt$$

where *Ct* represents residue level (μg g^−1^) at time *t* (days), is the dissipation rate constant presented as *K* and *C*_0_ is the initial deposit (μg g^−1^) at zero time (day of final application).

The half-life (DT_50_) period in days of applied imidacloprid in capsicum was estimated using the equation

$${\text{DT}}_{50}=\ln\;2/K=0.693/K$$

where, *K* is the gradient of the regression line which expresses as the speed of dissipation.

#### 2.10.2 Dietary risk assessment

The residual data produced during crop growing season under polyhouse and open conditions were used to estimate the dietary risk assessment of imidacloprid on capsicum crop. In order to understand the intake risk of imidacloprid residues in capsicum fruit, we estimated the dietary risk by utilizing the hazard quotient (HQ) value. Hazard quotient (HQ) value was derived by the following equation: HQ = EDI/ADI, where ADI and EDI represents, Acceptable daily intake and Estimated daily intake, respectively. EDI was estimated by using equation: (CRL × FI)/Average body weight where, CRL is the calculated residue level (mg kg^−1^) found in capsicum sample, FI is the food intake *i.e.,* the average daily food consumption (kg) which was recommended as 300 g and the average Indian adults body weight was considered as 60 kg [22] respectively. The respective ADI value for imidacloprid is reported as 0.006 mg kg^−1^body weight day^−1^ [23]. The HQ value if found higher than 1 (HQ > 1) indicated that the capsicum was contaminated with imidacloprid and there may be a chance of toxicity and unfit for human intake. Whereas, if the HQ value obtain less than 1 (HQ < 1) which means negligible hazard or safer to consumers consumption [24,25].

## 3 Results and discussion

### 3.1 Bio-efficacy of tested treatments against aphid infestation and yield evaluation in capsicum

**Fig. 1:**
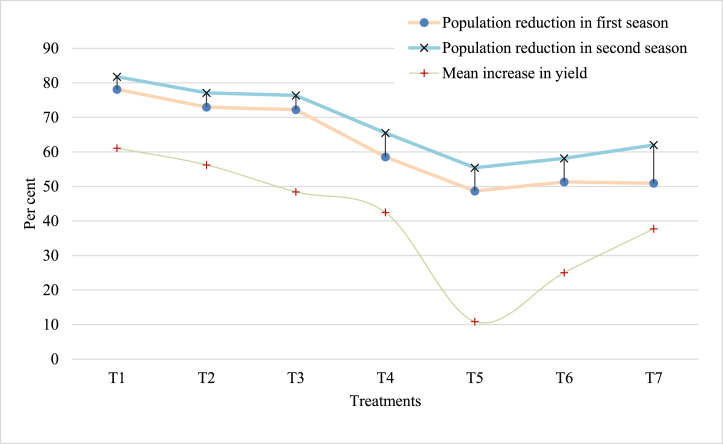
Bio-efficacy of insecticides against aphids and their effect on yield of polyhouse capsicum (T1 -Imidacloprid 17.8SL (Confidor®); T2 - Acephate 75SP (Starthene®); T3 - Dimethoate 30 EC (Rogor®); T4 - Buprofezin 25SC (Applaud®); T5 - *Metarhizium anisopliae*1.15%WP (1 × 108 cfu/ml) (Phule Metarhizium); T6 - *Lecanicillium lecanii*1.15%WP (1 × 108 cfu/ml) (Phule Bugicide); T7 - Neem oil-10000 ppm (Nimbicidine®)).

The bio-efficacy experiment conducted against the occurrence of aphids (*Myzus persicae*) infestation on capsicum crop before and after two sprays of treatments during two seasons under polyhouse is presented in Table 2 and their effect on yield is presented in Fig. 1. The results obtained from both seasons, the aphid population recorded a day before spraying (DBS) varied from 8.83 to 9.02 aphids/leaf, which indicated homogenous distribution of aphid population in the experimental plots. Among the tested insecticides and bio-pesticides against aphid infestation, imidacloprid 17.8SL significantly recorded a lesser number of aphids per leaf after two sprays (1.74 ± 0.06), accompanied by insecticides acephate 75SP and dimethoate 30 EC (2.17 ± 0.07 and 2.23 ± 0.07 aphids/leaf, respectively). Initially, a reduction in aphid populations was observed, and this reduction further increased 15 days after the initial spray. The treatments exhibited a decrease ranging from 56.43 % to 79.94 % compared to the control. Imidacloprid 17.8SL consistently maintained its effectiveness at 10 and 15 days after both the sprays (2.67 ± 0.04 and 4.97 ± 0.01 after first spray and 1.50 ± 0.11 and 1.74 ± 0.06 after second spray). Among bio-pesticides, the order of effectiveness was neem oil (3.78 ± 0.17/leaf) followed by *L. lecanii* (3.93 ± 0.17/leaf) and *M. anisopliae* (4.17 ± 0.11/leaf). The level of effectiveness recorded was similar over two seasons; however, it was found that two consecutive applications at 15 days gap are needed for the successful control. The population reduction of aphid was maximum in chemical insecticides *i.e.,* imidacloprid (79.94 and 80.39 %) followed by acephate and dimethoate which provided at par control of ∼75 % aphids population reduction.

**Table 2:** Bio-efficacy of insecticides against aphids infesting polyhouse capsicum.

Treatment details	Number of aphids per leaf							Population ROC (%)^b^	Population reduction over PTC (%)^b^
	PTC^a^	1st spray			2nd spray				
		7 DAS^b^	10 DAS^b^	15 DAS^b^	7 DAS^b^	10 DAS^b^	15 DAS^b^		
**T1 - Imidacloprid 17.8SL**	8.88 ± 0.07^a^	2.00 ± 0.06^f^	2.67 ± 0.04^e^	4.97 ± 0.01^de^	1.94 ± 0.04^e^	1.50 ± 0.11^d^	1.74 ± 0.06^d^	79.94^a^	80.39^a^
	(3.06)	(1.58)	(1.78)	(2.34)	(1.56)	(1.41)	(1.50)		
**T2 - Acephate 75SP**	8.83 ± 0.04^a^	2.25 ± 0.06^ef^	3.08 ± 0.05^de^	5.19 ± 0.01^cd^	2.30 ± 0.08^e^	1.77 ± 0.11^d^	2.17 ± 0.07^cd^	75.05^ab^	75.47^a^
	(3.06)	(1.66)	(1.89)	(2.39)	(1.67)	(1.51)	(1.63)		
**T3 - Dimethoate 30 EC**	8.90 ± 0.08^a^	2.35 ± 0.06^e^	3.17 ± 0.04^d^	5.23 ± 0.02^c^	2.32 ± 0.06^e^	1.75 ± 0.09^d^	2.23 ± 0.07^cd^	74.28^ab^	74.91^a^
	(3.07	(1.69)	(1.91)	(2.39)	(1.68)	(1.50)	(1.65)		
**T4 - Buprofezin 25SC**	8.98 ± 0.02^a^	2.55 ± 0.06^e^	3.33 ± 0.04^cd^	5.52 ± 0.02^b^	3.63 ± 0.30^d^	3.00 ± 0.31^c^	3.30 ± 0.11^bcd^	62.00^abc^	63.27^ab^
	(3.08)	(1.75)	(1.96)	(2.45)	(2.03)	(1.87)	(1.95)		
**T5 - *Metarhizium anisopliae***	8.97 ± 0.09^a^	7.57 ± 0.09^b^	6.57 ± 0.21^b^	4.80 ± 0.18^e^	4.70 ± 0.18^b^	4.03 ± 0.50^b^	4.17 ± 0.11^b^	52.02^bc^	53.53^b^
	(3.08)	(2.84)	(2.66)	(2.30)	(2.28)	(2.13)	(2.16)		
**T6 -*Lecanicillium lecanii***	9.02 ± 0.02^a^	7.02 ± 0.0^c^	6.20 ± 0.21^b^	4.42 ± 0.24^f^	4.30 ± 0.29^bc^	3.43 ± 0.56^c^	3.93 ± 0.11^b^	54.70^c^	56.38^b^
	(3.08)	(2.74)	(2.59)	(2.22)	(2.19)	(1.98)	(2.11)		
**T7 - Nimbicidine**	8.60 ± 0.01^a^	3.23 ± 0.08^d^	3.70 ± 0.04^c^	5.75 ± 0.01^b^	4.00 ± 0.33^cd^	3.55 ± 0.39^bc^	3.78 ± 0.17^bc^	56.43^c^	56.01^b^
	(3.02)	(1.93)	(2.05)	(2.50)	(2.12)	(2.01)	(2.07)		
**T8 - Untreated control (no spray)**	8.90 ± 0.06^a^	8.30 ± 0.11^a^	8.03 ± 0.04^a^	8.30 ± 0.07^a^	8.58 ± 0.03^a^	8.52 ± 0.03^a^	8.68 ± 0.03^a^	–	–
	(3.07)	2.97)	(2.92)	(2.97)	(3.01)	(3.00)	(3.03)		
S.Em.	0.187	0.112	0.138	0.087	0.172	0.186	0.557	6.319	5.759
LSD	NS	0.338	0.418	0.264	0.523	0.566	1.690	19.473	17.747
CV	3.647	4.381	5.197	2.730	7.525	9.400	25.742	16.918	15.181

**Note:** DAS: days after spraying, ROC- Reduction over control.

Figures in the parentheses are √(x+0.5) transformed values of original values. S.Em.: standard error of mean.CV: Coefficient of variation. All values with the same superscript, either a, b, c, d, e or f, are statistically similar; however, different superscripts are significantly different.

a PTC-Pretreatment count, which found non-significant at p = 0.05.

b Indicates the level of significance at p < 0.001.

The harvested fruits were weighed and extrapolated the data to yield per hectare is presented in Table 3. The data revealed that the cumulative yield in all the treatments existed considerably higher than untreated control (13.40 t/ha). Insecticide imidacloprid 17.8SL treated plots produced maximum yield of 34.43 t/ha over control (61.09 %) followed by acephate 75SP (30.61 t/ha with 56.24 % increase) and dimethoate 30 EC (25.98 t/ha with 48.43 % increase). In the order, the next best treatments with higher yield were buprofezin 25SC (23.30 t/ha with 42.50 % increase), nimbecidine (21.51 t/ha with 37.73 % increase), *L. lecanii* (17.87 t/ha with 25.02 % increase) and *M. anisopliae* (15.03 t/ha with 10.88 % increase). The ANOVA performed on yield data, and it was found that there were significant yield differences due to the chemical treatment applications. To know the pair-wise performances among the treatments, LSD was performed. It is clear from Table 3 that six treatments have significant effects on yield production, except T5, over the control treatment. The cost effectiveness (C:B ratio) of different treatments were ranged from 1.95 to 4.47 with the highest C:B ratio of 1:4.47 being recorded in imidacloprid 17.8SL and was accompanied by acephate 75SP (1:3.97), dimethoate 30 EC (1:3.37) and buprofezin 25SC (1:3.02). However, among different biopesticides, higher C:B ratio was recorded in nimbecidine (1:2.78) followed by *L. lecanii* (1:2.32) and *M. anisopliae* (1:1.95).

**Table 3:** Cumulative yield and cost economics of capsicum in polyhouse under different treatments used against aphids infesting.

Treatment details	Yield (t/ha)***	Increase in yield over control (%)	Additional yield over control (t/ha)	C: B ratio
T1 - Imidacloprid 17.8SL	34.43^a^	61.09	21.04	1: 4.47
T2 - Acephate 75SP	30.61^b^	56.24	17.22	1: 3.97
T3 - Dimethoate 30 EC	25.98^c^	48.43	12.58	1: 3.37
T4 - Buprofezin 25SC	23.30^d^	42.50	9.90	1: 3.02
T5 –*Metarhizium anisopliae*	15.03^f^	10.88	1.64	1: 1.95
T6 –*Lecanicillium lecanii*	17.87^e^	25.02	4.47	1: 2.32
T7 - Nimbicidine	21.51^d^	37.73	8.12	1: 2.78
T8 - Untreated control (no spray)	13.40^f^	–	–	1: 1.74
S.Em.	0.792			
LSD	2.404			
CV	6.031			

S.Em.: Standard error of mean.CV: Coefficient of variation. All values with the same superscript, either a, b, c, d, e or f, are statistically similar; however, different superscripts are significantly different.*** indicates the level of significance at p < 0.001.

Note: The calculations were based on the market value of each input during 2018: Imidacloprid 17.8SL – Rs. 1419 per 500 mL; Acephate 75SP – Rs. 72 per 100 mL; Dimethoate 30 EC – Rs. 122 per 250 mL; Buprofezin 25SC – Rs. 2000 per 1ltr; *M. anisopliae –* Rs. 200 per 1 Kg; *L. lecanii -* Rs. 200 per 1 Kg; NSE 5 % - Rs. 1458 per 1ltr; Capsicum – Rs. 75,000/- per tonne (market rate −2018); Cost of labour – Rs. 3000/- (500 × 6); Total cost of cultivation except plant protection measures – Rs. 5,74,329/-

The statistical analysis was performed on the data sets described above. ANOVA of RBD was carried out to identify the effects of different treatments over different stages. Results showed that when the performances among the seven chemical treatments were studied on PTC, it was found that there were no significant differences in aphid population. Later, when the performances among the seven chemical treatments were studied, it was also found that all the seven chemical treatments resulted in significant reduction of aphid populations over the control. In some cases, the efficiency performance of some chemicals was found at par with others. Hence, the LSD comparison was performed for comparing the treatments and their grouping was done. The results have been incorporated in Table 2. Also, it was found that there was high significant difference on the aphid population between PTC and on 15 days after 2nd spray as probability value (p-value) of *t*-test statistic (t-cal = 6.67) obtained as p < 0.001. Similar tests were also conducted to know whether there was significant difference on the aphid population reduction between the spray of chemicals at different interval days and non-significance results were obtained. Moreover, the effect of chemical treatments on population ROC (%) as well as on population reduction over PTC (%) were studied and found that all chemical treatments have significant effects.

Similar observations were reported [26] at the treatment dose (30 g a.i.ha^−1^) of imidacloprid 17.8SL provide the best management against the aphids population reduction (74.4 %) on okra above the untreated plot. Lowest number of aphid population in capsicum crop with applications of imidacloprid (0.025 % and 0.05 %) was reported [27]. Applications of imidacloprid 17.8SL at 22.5 g a.i. ha^−1^ in chilli was reported as the most effective and significantly superior insecticide with reduction of around 90 % of aphid population with no phytotoxicity [28]. Similarly [29], the highest reduction of aphid population after two sprays of confidor (350SC) was recorded in chilli. The findings of [30,31]; [27,[31], [32], [33], [34]] also matched with our current findings they revealed that imidacloprid was the best insecticide which efficiently reducing the sucking pest infesting in different crops.

### 3.2 Method validation

**Fig. 2:**
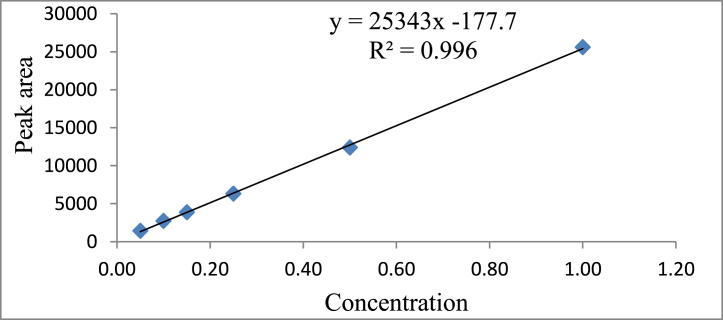
Calibration curve of imidacloprid standard.

The analytical method found a satisfactory value of correlation coefficient (R^2^) of 0.996 with the acceptable linearity range (0.05–1.0 μg/mL) mentioned in Table 4 and Fig. 2. LOD and LOQ values in this developed technique were found to be 0.015 and 0.05 μg/g in both matrices and the value was observed considerably lesser than the established maximum residue limit value of imidacloprid in the capsicum sample. The imidacloprid residue in capsicum was recognized by matching the sample peak retention time (RT) along the standard peak. Imidacloprid in the capsicum and soil samples were detected at retention time of 2.79 ± 0.10 min under the established method with acceptable specificity. The results of different method validation parameters of the established method were given in Table 5. Recovery percentages of the insecticide imidacloprid from capsicum and soil matrices were obtained satisfactory as the recovery efficiency and relative standard deviation values were found in the suitable range in between 93.61 % to 95.59 % and 3.44–6.80 %, respectively. HorRat values represented the method reproducibility which were found in between the acceptable range of 0.55–0.86 [35]. Matrix effect values were also found satisfactory ranges in between 94.19 and 96.59 % [17]. The process efficiency (PE) of the developed method was observed very much promising and the values were found >88 %. The current analytical method thus satisfies the benchmarks mentioned in the guidelines [17] and it has been found very much effective for the residue estimation of imidacloprid in capsicum crop and their field soil.

**Table 4:** Linearity, Retention time and LOD, LOQ value of analytical standard imidacloprid.

Parameter	Imidacloprid standard
Calibration range (μg/mL)	0.05–1.0
Calibration equation	y = 25343× - 177.7
Correlation coefficient (R^2^)	0.996
RT (min)	2.79
LOQ (μg/g)	0.05
LOD (μg/g)	0.015

Note: RT- Retention time; LOQ – Limit of quantification; LOD – Limit of detection.

**Table 5:** Method validation parameters of imidacloprid.

Compound	Substrate	Fortified level (mg/kg)	Recovered Residue (mg/kg)	SD %	RSD %	PRSD %	HorRat ratio	RE %	ME %	PE %
Imidacloprid	Capsicum	0.05	0.047	6.45	6.8	8.89	0.77	94.7	95.5	90.5
		0.1	0.095	5.04	5.3	8.00	0.66	95.1	96.1	91.4
		0.25	0.236	4.68	4.9	6.99	0.71	94.2	96.4	90.9
		0.5	0.478	3.29	3.4	6.28	0.55	95.5	96.6	92.3
	Soil	0.05	0.047	5.71	6.1	8.90	0.69	93.6	94.2	88.2
		0.1	0.095	4.62	4.8	8.01	0.61	94.5	95.8	90.5
		0.25	0.234	5.48	5.8	6.99	0.84	93.6	94.7	88.6
		0.5	0.470	5.11	5.4	6.30	0.86	93.9	95.1	89.3

Note: SD- Standard deviation; RSD - Relative Standard Deviation; PRSD - Predicted Relative Standard Deviation; HorRat - Horwitz Ratio; RE – Recovery Efficiency; ME - Matrix Effect; PE - Process Efficiency.

### 3.3 Persistence and dissipation studies of imidacloprid residues

The persistence and dissipation behaviour of imidacloprid on capsicum crop under open and polyhouse conditions are presented in Table 6. After the second spray, the initial deposits of imidacloprid residues in capsicum samples were measured 0.819 and 1.139 mg/kg under polyhouse and 0.607 and 1.023 mg/kg in open condition on both treatment dosages respectively. The residues of imidacloprid were dissipated very quickly under both conditions and found below detected level at 7 days after the second application of imidacloprid at recommended dose and at 10 days in case of double doses. It was found the residues of applied insecticide was dissipated to 91.7 % in less than 7 days at application doses of 25 g a.i. ha^−1^ and 93.1 % within 10 days when applied at @ 50 g a.i. ha^−1^ under a polyhouse. Whereas, in open condition pesticide residue was dissipated to almost 96.4 % in 5 days at recommended doses and at double doses pesticide was dissipated to 95.9 % within 7 days respectively. The dissipation pattern of imidacloprid in capsicum showed the first order kinetics with the correlation coefficient (R^2^) value of 0.989 and 0.994 under polyhouse and 0.961 and 0.984 in open condition at both treatment doses. The half-life period (DT_50_) of imidacloprid applied at 25 g a.i. ha^−1^ and 50 g a.i. ha^−1^doses was calculated with 1.88 and 2.66 days, in the polyhouse and 1.07 and 1.52 days in the open field. The soil collected from the crop field at harvest time did not had any imidacloprid residues.

**Table 6:** Persistency and dissipation of imidacloprid in capsicum crop under polyhouse and open condition.

Sampling Interval (days)	Polyhouse				Open field			
	Single dose (25 g a.i. ha^−1^)		Double dose (50 g a.i. ha^−1^)		Single dose (25 g a.i. ha^−1^)		Double dose (50 g a.i. ha^−1^)	
	Residue (mg/kg)	Dissipation (%)	Residue (mg/kg)	Dissipation (%)	Residue (mg/kg)	Dissipation (%)	Residue (mg/kg)	Dissipation (%)
0	0.819 ± 0.023	–	1.139 ± 0.027	–	0.607 ± 0.011		1.023 ± 0.011	–
1	0.549 ± 0.014	32.9	0.762 ± 0.016	33.1	0.388 ± 0.08	36.1	0.735 ± 0.07	28.1
3	0.288 ± 0.023	64.8	0.475 ± 0.009	58.3	0.153 ± 0.021	74.8	0.382 ± 0.05	62.7
5	0.106 ± 0.014	87.1	0.288 ± 0.011	74.7	0.022 ± 0.04	96.4	0.131 ± 0.010	87.2
7	0.068 ± 0.010	91.7	0.149 ± 0.013	86.9	nd		0.042 ± 0.04	95.9
10	nd		0.078 ± 0.010	93.1	nd		nd	
15	nd		nd		nd		nd	
Half-life (t1/2) days	1.88		2.61		1.07		1.52	
Regression equation	y = −0.160× - 0.094		y = −0.115x + 0.024		y = −0.282× - 0.139		y = −0.197x + 0.072	
Correlation coefficient (R^2^)	0.989		0.994		0.961		0.984	
Soil (harvest)	nd		nd		nd		nd	

*nd = Not detected.

The initial deposit of imidacloprid residue in capsicum fruits under polyhouse was observed little bit higher as compared to open field irrespective of the doses which could be due to the microclimate inside the polyhouse [36]. Almost similar result was reported by Ref. [37]), where initial deposit of pesticide residue in capsicum fruits was found 3.80 mg/kg under polyhouse and 2.47 mg/kg in open condition. Initial deposits of imidacloprid in okra fruits [38] was 0.89–3.52 mg/kg and half-lives (DT_50_) were reported 1.24–3.47 days @ 20, 40 and 80 g a.i. ha^−1^ treatment doses. The current findings of our studies were also matched with the results [39] who observed the half-lives (DT_50_) of imidacloprid was 2.8 days in cucumber in greenhouse. In brinjal crop residues of confidor insecticide was found up to 10 days after the applications of insecticide at 20 and 40 g a.i. ha^−1^ [40]. Similarly, the DT_50_ values of confidor (17.8SL) on chilli fruits was observed 1.41 and 1.65 days and in case of on chickpea pods the half-life period was observed 2.07 and 2.31 days while in chickpea leaves DT_50_ values were reported 1.75 and 1.72 days when applied at single and double of the treatment doses [41]. However, some other factors such as present weathered conditions, fruits size, crop variety, metabolism, chemical and photoreaction influenced the degradation and dissipation kinetics as well as, initial deposits and residues of imidacloprid in capsicum fruits [32,36,42]. Whereas the parameters like organic carbon, soil pH, soil moisture and soil microbial status influences the degradation of insecticides reached in soil systems [43,44].

### 3.4 Dietary risk assessment

The dietary risk assessment of imidacloprid was calculated by considering the national average daily vegetable intake, standard weight of the adult person and residues present in the samples of the current study. Estimated dietary intake (EDI) and hazard quotient (HQ) of imidacloprid in capsicum was presented in Table 7. The EDI values of the imidacloprid in capsicum fruits were found 0.0041 and 0.0057 mg kg^−1^bw day^−1^ under protected and 0.003 and 0.0051 mg kg^−1^bw day^−1^ in open condition at 0 days (initial deposit) which was far lower value than the established MRL value (0.5 mg/kg) of imidacloprid [45]. The HQ ranged from 0.06 to 0.68 and 0.07 to 0.95 in polyhouse where the value 0.02 to 0.51 and 0.03 to 0.85 were found in open field at both the treatment level, respectively. The dietary exposure of imidacloprid on capsicum fruit under both conditions exposed that HQ values obtained from the different treatment doses have not exceeded the upper limit of toxicity (HQ < 1). The imidacloprid residues in capsicum fruits were found below the existing MRL values (0.5 mg/kg) at 3 days after its final applications. As a result, imidacloprid residues present on capsicum fruits do not cause any harms for human intake.

**Table 7:** Dietary risk assessment of imidacloprid under polyhouse and open condition.

Sampling Interval (days)	Polyhouse				Open field			
	Single dose (25 g a.i. ha^−1^)		Double dose (50 g a.i. ha^−1^)		Single dose (25 g a.i. ha^−1^)		Double dose (50 g a.i. ha^−1^)	
	EDI (mg/kg bw/day)	HQ	EDI mg/kg bw/day)	HQ	EDI mg/kg bw/day)	HQ	EDI mg/kg bw/day)	HQ
0	0.0041	0.68	0.0057	0.95	0.0030	0.51	0.0051	0.85
1	0.0027	0.46	0.0038	0.63	0.0019	0.32	0.0037	0.61
3	0.0014	0.24	0.0024	0.40	0.0008	0.13	0.0019	0.32
5	0.0005	0.09	0.0014	0.24	0.0001	0.02	0.0007	0.11
7	0.0003	0.06	0.0007	0.12	–	–	0.0002	0.03
10	–	–	0.0004	0.07	–	–	–	–
15	–	–	–	–	–	–	–	–

Note: EDI - Estimated Daily Intake; HQ - Hazard Quotient.

## 4 Conclusion

The present study explained that imidacloprid was the most efficient insecticide for controlling aphid infestation in capsicum crop, through the maximum crop production of 34.43 t/ha and provided best C:B ratio of 1:4.47. However, among the non-chemical insecticides, nimbecidine (Botanical) and *L. lecanii* (Entomopathogen) were also found effective as compared to control under polyhouse condition. In addition, we explored the persistence behavior of imidacloprid in open field and under polyhouses on capsicum crop. A unique, cost effective, simple technique was validated by HPLC-PDA system for evaluation of imidacloprid deposit on capsicum fruit and soil. The result describes that imidacloprid dissipated quickly in capsicum, and soil under both conditions leaving very minute quantity of residues in the system with DT_50_ value of 1.88 and 1.07 days at recommended treatment level in polyhouse and open condition. Thus, suggesting that imidacloprid (17.8SL) applied at 25 g a.i. ha^−1^ may be recommended for the economical and effective management of aphid infestation in capsicum crop without any health problems to the consumer.

## Funding

The authors are thankful to the Mahatma Phule Krishi Vidyapeeth, Maharashtra, India for financial assistance.

## Data availability statement

All data that support the findings of the present study are included in the article.

## Ethics approval and consent to participate

Not applicable.

## Consent for publication

Not applicable.

## CRediT authorship contribution statement

**Guru Peelapura Ningarajappa:** Writing – original draft, Resources, Methodology, Investigation, Formal analysis, Conceptualization. **Chidanand Shiveshankar Patil:** Project administration, Formal analysis, Conceptualization. **Bhaidas Vitthal Deore:** Investigation, Formal analysis, Conceptualization. **Yogesh Subhash Saindane:** Methodology. **Rahul Kumar Anurag:** Resources, Methodology. **Ashish Kumar Singh:** Writing – review & editing, Writing – original draft, Software, Data curation. **Samir Barman:** Formal analysis. **Tilak Mondal:** Writing – review & editing, Writing – original draft, Software, Methodology, Formal analysis.

## Declaration of competing interest

The authors declare that they have no known competing financial interests or personal relationships that could have appeared to influence the work reported in this paper.

## Abbreviations

*BQL*: Below Quantification Limit
*a.i*: Active Ingradient
*CIBRC*: Central Insecticide Board and Registration Committee
*EFSA*: European Food Safety Authority
*nAChRs*: Nicotinic Acetylcholine Receptor
*RBD*: Randomized Block Design
*ETL*: Economic Threshold Level
*ROC*: Reduction Over Control
*PTC*: Pre-Treatment Count
*QuEChERS*: Quick, Easy, Cheap, Effective, Rugged and Safe
PSA: Primary Secondary Amine;
GCB: Graphitic Carbon Black
RP-HPLC: Reverse Phase High Performance Liquid Chromatography
RE: Recovery Efficiency
ME: Matrix Effect
PE: Process Efficiency
RSD: Relative Standard Deviation
PRSD: Predicted Relative Standard Deviation
LOD: Limit of Detection
LOQ: Limit of Quantification
HQ: Hazard Quotient
ADI: Acceptable Daily Intake
EDI: Estimated Daily Intake
CRL: Calculated Residue Level
FI: Food Intake
DBS: Day Before Spraying
